# 

**Published:** 1897-10-02

**Authors:** 


### The Hospital

A JOURNAL OF

THE MEDICAL SCIENCES AND HOSPITAL ADMINISTRATION,

INCLUDING

A SPECIAL SECTION FOR NURSES.

55

EDITED BY

SIR HENRY BURDETT, K.C.B.,

AND

SOLOMON C. SMITH, M.D.

IN TWO VOLUMES ANNUALLY.

SURGEON GENL'S OFFICE. 165460 LIBRARY.

VOLUME XXIII.

October, 1897, to March, 1898.

London:
PUBLISHED BY THE SCIENTIFIC PRESS (LIMITED), AT THE HOSPITAL BUILDING,
28 & 29, SOUTHAMPTON STREET, STRAND, W.C.

MDCCCXCVIII.

W1 H069X

### INDEX TO VOL. XXIII., 1897—8.

*** Articles under the general heading *Medical Progress and Hospital Clinics* are distinguished by the initial (C.) for *Hospital Clinics*, and by (P.) for the rest of the series. (W.) distinguishes the *Institutional Workshop* articles.

Abdominal Contusions with Visceral Lesions (P.), 275
Abdominal Cystic Tumours (P.), 275
Abdominal Section, The Patient Insisted upon Undergoing, 412
Abdominal Surgery (P.), 11
Abortion (P.), 81
Abortion-mongers, 389
Abscess, Hepatic (P.), 331
Abscess of the Liver (P.), 65
Abscess, Subphrenic (C.), 430
Adelaide Hospital, The, 305
Albuminuria and Eclampsia in Pregnancy (P.), 119
Alcohol and Cirrhosis, 234
Alcohol, The Action of, in Acute Disease, 309
Amblyopia, Toxic (P.), 279
Anæsthetics, Deaths from (C.), 310, 328, 342
Anæsthetics, Two Points in Administering (C.), 430
Anæsthetics, Vomiting While Under (C.), 44
Anastomosis, Intestinal (P.), 431
Angina Pectoris, Cardiac Pains and (C.), 116, 132
Angioma, Cavernous, of the Spleen (P.), 451
Ante-partum Hæmorrhage (P.), 99
Antiseptics (P.), 205
Anti-toxin, Diphtheria in Epidemic Measles (C.), 274
Anti-Vaccinators, A Short Way with, 302
Anus, Rectum and (P.), 46, 433
Aorta, Perforation of the, by Calcareous Tracheal Glands (C.), 79
Appendicitis (P.), 172; Acute, 11; Classification of, 11; Experimental, 11; Pre-diagnostic Treatment, 11; Treatment, 11
Are we quite sure that we are right? 389
Argonin. *See p.* 330
Around the Hospitals, 109
Artificial Lighting (P.), 293
Asepsis (P.), 257
Asepsis v. Antisepsis (P.), 330
Astringents in the Treatment of Eye Disease (P.), 330
Asylum Construction (W.), 176, 192
Asylum Worthies (W.), 366
Australia, Notes from (W.), 350
Bacteria in the Blood (P.), 44
Baldness, Seborrhœa and (P.), 189
Barry, The Death of Dr., 36
Bee-stings, Immunity from (C.), 378
Berbice Colonial Lunatic Asylum (W.), 230
Berbice County Hospital, New Amsterdam (W.), 211
Beri-beri (P.), 256
Bicycling for Women (P.), 155
Bile Ducts, Infective Inflammation of the (P.), 82
Bile Ducts, Injuries to the (P.), 83
Bile Ducts, Tumours of the (P.), 83
Bladder (P.), 364
Bladder, Cystic Tumours of the (C.), 117
Blood, Diseases of the (P.), 9, 413
Blood-letting, 37
Bognor, Children's Convalescent Home, Laying the Foundation Stone (W.), 102
Boils, Carbuncles, and Whitlows (C.), 44
Book World of Medicine, The (*Books, Magazines, and Pamphlets reviewed or noticed, and articles relating to*):—
Aldersmith. Ringworm and Alopecia areata, 175
Allbutt (edited by). A System of Medicine, 261
Bashore. Outlines of Rural Hygiene, 453
Bice and Crothers. Elements of Latin, 453
Broadbent. Heart Disease, 123
Broca and Lubet-Bardon (trans. by Curtis). Mastoid Abscesses and their Treatment, 261
Brown. Economics, Anæsthetics, and Antiseptics in Midwifery, 123
Cross. Health in Africa, 191
Crothers. *See Bice*
Curtis. *See Broca*
Dowse. The Pocket Therapist, 283
Eyre. *See Mendini*
Firth. *See Notter*
Fletcher. Commercial Uses of Coal-gas, 191
Freyberger. Pocket Formulary for the Treatment of Disease in Children, 453
Giles. *See Sutton*
Gordon. Sir James Young Simpson and Chloroform, 347
Green. Memory and its Cultivation, 157
Greene. *See Lippincott*
Guide to Bath, Bristol, &c., 453
Hart (edited by). Masters of Medicine (series), 49, 207
Hart and Hendley. Sanitation and Health, 139
Hendley. *See Hart*
Hewetson. Localisation of Headache, 283
Hutchinson and Rainy. Clinical Methods, 49
Lippincott's Medical Dictionary (edited by Greene), 49
London, Annual Report of the Medical Officer of Health of, 347
Book World of Medicine (*continued*).
Lubet-Bardon. *See Broca*
Macdougall. *See Rhodes*
McKay. Lawson Tait's Perineal Operations, &c., 191
Maclaren. A Doctor of the Old School, 417
Madden. Notes on the Special Hygiene of Childhood and Health, 123
Meadowcroft. A B C of the X-rays, 13
Medical Annual 1898, 435
Mendini (trans. by Eyre). Hygienic Guide to Rome, 67
Mitchell. Fat and Blood, 417
Moor. *See Pearmain*
Norris and Oliver. System of Diseases of the Eye, 13
Notter and Firth. Practical Domestic Hygiene, 157
Oliver. *See Norris*
Paget. John Hunter, 49
Pearmain and Moor. Analysis of Food and Drugs, 227
Poore. Nervous Diseases of the Hand, 365
Poore. The Dwelling House, 157
Power. William Harvey, 207
Rabagliati. On Food and Exercise, 207
Rainy. *See Hutchinson*
Reynolds. Cairo of To-day, 417
Rhodes and Macdougall. Treatment of Imbeciles and Epileptics, 435
Roberts. Spas of Wales, 29
Schaeffer. Anatomical Atlas (Obstetrics), 29
Schaeffer. Atlas and Essentials of Gynæcology, 29
Scholtz. South African Climate, 283
Scott. Manual of Urine Testing, 283
Shadwell. The Tallerman Treatment by Superheated Dry Air, 435
Sibley. Local Hot Dry Air Treatment in Acute and Chronic Gout, 191
Smith. Spinal Caries, 139
Starling. Elements of Human Physiology, 49
Stedman (Edited by). Twentieth Century Practice of Medicine, 365
Sutherland. The Insane in Private Dwellings and Licensed Houses, 67
Sutton and Giles. Diseases of Women, 227
Thin. Psilosis or "Sprue," its Nature and Treatment, 297
Thompson. Mystery and Romance of Alchemy and Pharmacy, 139
Transactions of the British Institute of Preventive Medicine, 417
Vincent. Elements of Hypnotism, 175
Wade. Introduction to the Study of Organic Chemistry, 297
Walsh. Premature Burial; Fact or Fiction, 435
Walsh. The Röntgen Rays in Medical Work, 67
Wharton. Practice of Surgery, 297
Bradford Royal Infirmary Nurses' Home (W.), 349
Brain Fatigue in School Work, 323
Brain, Surgery of the (P.), 312
Brain, Tumours of the (P.), 295
Breech Presentation (P.), 100
British Laryngological, Rhinological, and Otological Association (C.), 134, 359
British Medical Journal, The, 305
British Orthopædic Society (C), 118
Cæsarian Section (P.), 119
Cagney, The Late Dr., 167
Calculi, Urinary (P.), 364
Calculus (P.), 45; Vesical (P.), 65, 380
Cancer (P.), 98
Cancer of the Breast, The Debate on (C.), 393
Cancer, Modern Operations for, 304
Cancer, Oophorectomy for (C.), 43
Cancer, Operations for Breast (C.), 359
Cancer of the Rectum (P.), 433
Carbuncles (C.), 44
Cardiac Pains and Angina Pectoris (C.), 116, 132, 150, 168
Catching Cold, 235
Cerebellar Operations, Chloroform in (P.), 296
Cerebral Localisation (P.), 312
Cerebral Surgery, Technique of (P.), 257
Cerebral Tumour, Optic Neuritis in (P.), 343
Cerebral Tumours, Operative Procedure (P.), 296
Certificates of the Cause of Death, 441
Chalfont St. Peter Homes for Epileptics (W.), 228, 319
Charing Cross Hospital (W.), 125
Charity Mendicants, 197
Chelsea Infirmary Nurses' Home (W.), 263
Children's Hospital in Paris, A (W.), 50
Chilly Churches, 37, 71
Chinese Laundrymen, 19
Chloroform and Polemics (C.), 413
Chloroform, Deaths from (C.), 170
Chlorosis (P.), 414
Cholangitis, Infective and Suppurative (P.), 346
Cholecystectomy (P.), 345
Cholecystenterostomy (P.), 83, 345
Cholecystitis (or Suppurative Catarrh) of the Gall Bladder (P.), 82
Cholecystostomy (P.), 345
Cholelithiasis (P.), 331
Chorea (P.), 343
Chorea, Habit (P.), 343
Chorea, Localising Factors in (C.), 78
Chorea, Minor Treatment of (P.), 344
Christmas Day, The Hospitals on (W.), 248
Circulatory Objects, Loss of Vision of (P.), 330
Cleanliness, 90
Clinical Society, The (C.), 43, 97, 133, 169
Coast Hospital, Little Bay, Sidney, The (W.), 265
Conjunctivitis, A Case of Diphtheritic (P.), 294
Constipation and the Corset (C.), 291
Constrictions, Multiple, of Tubercular Origin (P.), 431
Contracted Pelvis, Delivery in (P.), 99
Convict on the Road, The, 373
Cornea, Ulcers of the, Treatment of, in Children, (C.), 222
Corneal Ulceration, Cautery in (P.), 280
Creasote as an Internal Antiseptic (C.), 396
Cycling as a Cause of Eye Worry (P.), 330
Cystitis in Paraplegia (C.), 378
Cystoscopy, Electric (P.), 65
Cysts, Hydatid (P.), 66
Cysts of the Kidney (P.), 45
Cysts, Mesenteric (P.), 415
Cysts, Pancreatic (P.), 452
Darenth Scandal, The, 321
Defæcation, The Mechanics of (C.), 118
Dermatology, Periscope of (P.), 137, 156
Devizes Hospital Collection (W.), 213
Diamond Jubilee and the Hospitals, The (W.), 298, 315
Digestive Organs, Diseases of the (P.), 97, 154, 172, 450
Dilatation of the Stomach (P.), 98
Diphtheria, The Carriers of, 75
Diphtheria, The Diagnosis and Treatment of (C.), 153
"Dispensaries," The Legal Control of, in New York, 300
Drugs, The Combination of Antagonistic (C.), 221
Drunk or Dying, 287, 318
Dublin, City of, Hospital, 109
Dundee Royal Infirmary, 109, 305
Duodenum, Ulcer of the (P.), 187
Dysentery (P.), 187
Dysmenorrhœa (P.), 135
Ear Disease and "Head Symptoms" in Infants (C.), 180
Ear, Pain in the (C.), 392, 410
Eclampsia, Albuminuria and, in Pregnancy (P.), 119
Ectopic Gestation (P.), 82, 99
Eczema (P.), 156
Editor's Letter-box, 71, 142, 161, 178, 248, 265, 283, 302, 318, 334, 381
Education and Crime, 165
1897: A Retrospect, 237
Enteric Fever, The Prevention of (C.), 201
Epilepsy (P.), 312, 362
Epilepsy, The Surgical Treatment of Focal (P.), 120
Epileptics, Homes for (W.), 228
Eruptions produced by Drugs (P.), 190
Erythromelalgia (P.), 363
Ether followed by Chloroform, 373
Eucain and Holocaine (P.), 330
Excess of Sanitary Zeal, 373
Expanding Bullets, 317
Expert Witnesses, 423
Exploratory Incisions, The Curative Influence of (C.), 411
Extra-Uterine Gestation (C.), 326
Extra-Uterine Pregnancy, The Frequency of (C.), 412
Extro-version of the Bladder (P.), 380
Eye Worry, Cycling as a Cause of (P.), 330
Fever Hospitals, The Extravagance of, 406
Fevers (P.), 203, 223, 242, 256, 311
Fever, Pernicious Malarial, at Sierra Leone (C.), 79
Fight: "A Finish Fight," 269
Filtration, The Curiosities of, 75
Final Touch, A, 235
Fistula in Ano (P.), 433
Fistula, Vesico-Vaginal (P.), 156
Formalin in Eye Disease (P.), 330
Fractures and Dislocations (P.), 225, 243, 257
Free Speech at Medical Societies (C.), 185
French Milk, 443
Fresh Air for the Cows, 389
Frozen Meat, Inspection of, 129
Gall-bladder. *See also Liver.*
Gall-bladder, Chronic Catarrh of the (P.), 82

Gall-bladder, Fistula of (P.), 346
Gall-bladder, Tumours of the (P.), 346
Gall-bladder and Bile Ducts, Ulceration, Perforation, Stricture, Tumours (P.), 83
Gall-stone Operations, Statistics of (P.), 83
Gall-stones, Intestinal Obstruction from (P.), 83
Gall-stones, "Surgical Significance of" (P.), 83
Gastric Catarrh (P.), 98
Gastric Ulcer (P.), 97, 276
Gastro enterostomy (P.), 277
Gastrostomy (P.), 276
General Medical Council, The (O.), 170
General Medical Council, The New Member of the, 57
Genito-Urinary Surgery (P.), 45, 380, 397
Genito-Urinary Surgery, Cases and Observations (P.), 65
George Town, British Guiana, Poor House at (W.), 52
Gestation, Extra-uterine (O.), 326
Glasgow Western Infirmary, The, 111
Glycerinated Vaccine Lymph, 215
Gonorrhœa (P.), 398
Gout, Rheumatism and (P.), 26, 62
Great Northern Central Hospital, 131
Greene, Mr. Richard, F.R.C.P.Edin. (W.), 366
Grosvenor Hospital for Women and Children (W.), 142
Growth and Scope of Voluntary Hospitals in London (W.), 14, 30, 51, 69, 140, 159, 262
Guardians and Operation Fees. See p. 142
Gynæcology in General Practice (O.), 134
Hæmoptysis (P.), 202
Hæmorrhage in Cranial Surgery (P.), 258
Hart, Ernest, D.C.L., M.R.C.S., An Appreciation, 281
Hart, Mr. Ernest, Illness of, 3; death of, 268
Harveian Oration, The (O.), 60
Harveian Society (O.), Creasote in Phthisis, 97
Head Injury, Clinical Lecture on Four Cases of (O.), 7
Headache (P.), 396
Hearing, The Mental Side of the Sense of (O.), 294
Heart, On the Investigation of some Nervous Diseases of the (O.), 40
Heart, Posture in Percussion of the (O.), 291
Heart, Wounds of the, 216
Hepatic Abscess (P.), 331
Heredity in Regard to Length of Life (O.), 395
Hernia (P.), 27, 63
Hernia, Rare Forms of (P.), 64
Hernia, Strangulated Cæcal (P.) 64; Umbilical (P.), 63
Herpes Zoster and Facial Paralysis (P.), 397
Hill, Mr. Leonard, on Dr. Lauder Brunton (O.), 448
Hints to Practitioners (O.), 292, 310
Holocaine and Eucain (P.), 330
Homœopathic Hospital, London (W.), 68
Hospital Administration (W.), 50
Hospital Construction (W.), 142, 158, 177, 211, 212, 228, 263
Hospital, Cost of a. See p. 53
Hospital Festivals, Meetings, &c. (W.), 144, 193, 208, 282, 333, 350, 368, 383, 400, 420, 439, 454
Hospital Fund, Prince of Wales's, 236
Hospital Reform (W.), 53, 73, 127, 141, 161
Hospital Reform: A Reverie (W.), 317
Hospital Reform Association (W.), 86, 301, 385
Hospital Reform, Facts and Principles as to, 128
Hospital Sunday Fund, Metropolitan (W.), 101
Hospital Sunday Fund, Metropolitan, and Street Collections (W.), 108
Hospitals, see Growth and Scope
Hospitals Association (W.), 103
Hospitals, Buildings and Fittings in the Older (W.), 245
Hospitals and Their Detractors (W.), 176
Hospitals, General and Infectious Diseases, 32
Hospitals and Life Governors, 251
Hospitals and Vivisection, 303
Hydronephrosis (P.), 45
Hypnotism, The Physiology of, 129
Hysterectomy, A Very Complete (O.), 449
Hysteria, On Two Cases of (O.), 200
Hysterical Patients, Operations on (O.), 450
Identification of Children at Fever Hospitals (W.), 264
Infection by Third Persons, 164
Infectious Disease, Exemptions from the Duty of Notifying, 355
Infectious Diseases, The Diminishing Mortality from (O.), 274
Influenza (P.), 203, (O.), 241
Influenza in Children (O.), 291
Insane, Operations on the, 2
Insane, The Responsibility of the, 285
Insane: The Story of the Insane from Year to Year, 31, 176, 192, 213, 350, 404, 421
Intestinal Obstruction (P.), 416
Intestinal Operations, Statistics of (P.), 415
Intestines, Surgery of the (P.), 415, 431
Introductory Addresses (October, 1897), 23
Iris, Tubercle of the (P.), 280
Isolation Hospitals. See p. 248
Isolation Nurses for District Work, 197
Jerk, Crossed Abductor (P.), 364
Judges and Abusive Language, 407
Kidney, Cysts of the (P.), 45
Kidney, Granular and Vascular Changes (O.), 412
Kidney, Movable (P.), 45
Kidney, Rupture of the (P.), 45 (O.), 169
Kidney, Tuberculosis of the (P.), 45
Kidney, Two Cases of Operation on the (O.), 210
Kidneys and Ureters (P.), 364
Kindergarten and Eyesight (P.), 330
Knee Joint, Perforating Wounds of the (O.), 448
Knock-out Blow, A (O.), 203
Koch's New Tuberculin in the Treatment of Phthisis (P.), 186
Lady Doctors in Germany, 327
Laminectomy in Spinal Caries (P.), 188
Lamp Accident Season, The, 129
Larynx, Blocking of the, by a Caseous Gland (O.), 378
Leeds, A Lying-in Hospital for, 36
Leicester County Infirmary, The (W.), 124
Length of Life, Heredity in regard to (O.), 395
Lepers' Home, A (W.), 265
Leukæmia (P.), 413
Libel Actions by Medical Practitioners, 353
Lipomata, Retro-perineal and Perineal (P.), 275
Liver (Diseases of the Digestive Organs) (P.), 172
Liver, Abscess of the (P.), 65
Liver, Diagnosis of Neoplasms of the (P.), 331
Liver, Movable (P.), 331
Liver, Partial Resection of the (P.), 66
Liver, Operations on the (O.), 428, 446
Liver, Penetrating Wounds of the (P.), 331
Liver, Resection of the (P.), 331
Liver, Gall-Bladder and Ducts, Surgery of the (P.), 65, 82
Locomotor Ataxy (P.), 362
London Air, 425
London School Board, The, 55
London University, The Proposed, 405
London, University of, The Reorganisation of the (O.), 309
London Water Again, 251
Lumbar Puncture (P.), 363
Lungs, Diseases of the (P.), 173, 186, 202, 224
Lupus (P.), 137
Maidstone Epidemic, The (O.), 255, 327
Manchester Royal Infirmary, 287, (W.), 382
Marine Hygiene (P.), 278
Marriage and the Race, 3
Massage by the Blind, 113
Mastoid, Operations on the (P.), 278
Measles, Diphtheria Anti-toxin in Epidemic (O.), 274
Measles, The Prevention of, 318
Measles, The Treatment of, "a Mere Matter of Routine," 267
Measles, The Treatment of, by Nurses (O.), 327
Measles-Infected Children, Hospitals for (O.), 153
Medical Acts, Reform of the, 250
Medical Education, A Defect in, 17
Medical M.P.'s at Work, 18
Medical Session, The, 22
Medical Society, The (O.), 78, 118, 152
Meningitis (P.), 277; Acute Purulent (P.), 278; Simple Basic, in Infants (O.), 79
Mental Nursing, 265
Mercury, Intra-Muscular Injections of, in the Treatment of Syphilis (O.), 377
Mesenteric Cysts (P.), 415
Mesenteric Tumours, Solid (P.), 416
Microbes and Internal Medication (O.), 61
Midwife Difficulty in New York, The (O.), 396
Midwives, The Regulation or the Abolition of, 354
Milk Diet (O.), 185
Milk Laboratories, 222
Milk Supply, A Municipal, 181
Modern Sociology, 5, 21, 39, 59, 77, 93, 115, 131, 149, 167, 183, 199, 219, 253, 271, 289, 307, 325, 338, 357, 375, 391, 409, 427, 445
Monthly Reviews, The Degeneration of, 19
Mother in the Factory, The, 445
Mother, The Modern, 407
Mothers of England, 19
Motherhood and Militarism, 337
Multiple Constrictions of Tubercular Origin (O.), 431
Muzzling or Rabies, 195
Myopia, Extraction of Transparent Lens in Extreme (P.), 294
Nasal Polypi and their Treatment (O.), 341
Nasty, Wasteful Habit, A, 57
Nature's Cure for Over Population, 91
Nerves, Ascending Degeneration in Mixed (P.), 363
Nervous System, Diseases of the (P.), 396
Neuritis, Optic, in Cerebral Tumour (P.), 343
Neurology (P.), 343, 362
Neuron, The, and Disease (O.), 78
New Appliances and Things Medical, 48, 66, 84, 122, 138, 174, 206, 226, 244, 260, 280, 296, 314, 332, 346, 364, 416, 434, 452
Night Caps, 57
Notes (W.), 382, 400, 419, 438, 454
Notes and News, 4, 20, 38, 59, 76, 92, 114, 130, 148, 166, 182, 198, 217, 236, 252, 270, 288, 306, 324, 338, 356, 374, 390, 408, 426, 444
Notification Act, The, 184
Nursing in Rural Districts (W.), 437
Obstetrics (P.), 81, 99, 119, 135, 155
"Office," The Physician's, 217
"Official," or "Unofficial," 322, 381
Once for All, 147
Oophorectomy for Cancer (O.), 43
Operations, "Complete" and "Incomplete" (O.), 359
Ophthalmia, Gonorrhœal, Treated with Argonin (P.), 330
Ophthalmia, Treatment of Catarrhal (P.), 329
Optic Neuritis in Cerebral Tumour (P.), 343
Orphol (P.), 47
Out-Patient Problem, The (W.), 85, 87
Ovariotomy, Successful, in a Child Four Months Old (O.), 412
Pancreas, Spleen and, Surgery of the (P.), 451
Pancreatic Cysts (P.), 452
Pancreatitis (P.), 452
Park Hospital, The, 212
Park Hospital, Opening of the (W.), 158
Pathological Society, The (O.), 79
Pauper's Last Possession, The, 235
Payment of Jurymen in Coroners' Courts, 91
Péan, Dr., of Paris, The Death of, 327
"Peculiar People," The, 89
Pelvic Hæmatocele (P.), 155
Pemphigus (P.), 190
Peritoneal Cavity, Drainage of the (P.), 260
Peritoneum, Surgery of the (P.), 259
Peritonitis (P.), 259, Tuberculous (O.), 151, 184, 260
Phagedæna, The Bath Treatment of (O.), 396
Pharmacopœia, The New, 283
Philanthropy, The Economics of (W.), 349, 367, 399, 418, 436
Phthisis (P.), 186
Phthisis and other Diseases, Health Resorts for (P.), 2[illegible]4
Phthisis, The Climatic Treatment of (O.), 6
Phthisis, The Open-air Treatment of, in England (O.), 413
Plague, The (P.), 256 (O.), 327
Plague, The, in India, 371
Plague, The Bacillus of (O.), 309
Plague, The Incidence of, in Different Classes (O.), 256
Plague, The Recrudescence of the, in Bombay, 287
Playfair (Dr.) on Mediævalism at the Royal Colleges (O.), 447
Pleuritic Effusion, Serous, Treated by Incision (O.), 378
Pneumonia (P.), 173
Pneumonia, A Case of, with Cerebral Complications (O.), 340, 358, 375
Pneumonia, An Abnormal Case of, 429
Polypi, Nasal, and their Treatment (O.), 340
Poor House, A Tropical (W.), 52
Poor Man's Home, The, 5, 21, 39, 59, 77, 93, 115, 131, 149, 167, 183, 199, 219, 253, 271, 289, 307, 325, 338, 357, 375, 391
Post-Graduate Teaching in London, 181
Post-Mortem Examinations on Hospital Patients (W.), 229
Potts' Curvature of the Spine, Forcible Straightening in (O.), 133, 169
Potts' Disease, Immediate Reduction of the Deformity (P.), 88
Practical Departments (W.), 161
Practical Points (W.), 53
Pregnancy, Albuminuria and Eclampsia in (P.), 119
Pregnancy with Fibroids (P.), 119
Pregnancy with Ovarian Tumour (P.), 119
Prince Alfred Hospital, Sydney (W.), 177
Prince of Wales's Hospital Fund, 149, 230, 237
Prison Training and Administration, 409, 427
Private Clinic, The (O.), 449
Professional Reticence, The Advantages of, 407
Projectiles Used in the Græco-Turkish War, 217
Prosperity and Cancer, 193
Prostate (P.), 397
Purpura Hæmorrhagica (P.), 27
Pyloric Obstruction of Hepatic Origin (P.), 346
Pyoktanin (P.), 47
Pyo-pneumothorax, The Treatment of, 152
Quack Medicines, 335
Quain, The Late Sir Richard, 424
Quarantine, Shot Gun, 75
Quarantine, The Recrudescence of, 249
Queen's Speech, The, 337
Rabies (P.), 312
Rabies, The Prevention of (O.), 264
Radiant Heat for the Relief of Pain (O.), 431
Reciprocity in Medicine (O.), 171
Rectum and Anus (P.), 46, 433
Rectum as a Reservoir, The (P.), 433
Rectum, Cancer of the (P.), 433
Rectum, Prolapse, &c., of the (P.), 433
Rectum, Stricture of the (P.), 46
Redditch, The Smallwood Hospital (W.), 15
Reichman's Disease (P.), 98
Renal Circulation (P.), 45
Renal Disease in Relation to Surgical Operation (P.), 45
Renal Medicine (P.), 328, 361, 379
Retention and Extravasation of Urine in Children (O.), 431
Rheumatism and Gout (P.), 26, 62
Rheumatism, Localising Factors in (O.), 78
Rheumatoid Arthritis (P.), 26
Right to Practise, The, 355
Rods and Cones, Functions of the (P.), 280, 294
Röntgen Rays, The, 323
Röntgen Society, Inauguration of the (W.), 125
Royal British Nurses' Association, 145
Royal College of Surgeons of England, The (O.), 61
Royal Medical and Chirurgical Society, The (O.), 79, 117, 201

St. Anne's-on-Sea Convalescent Home (W.), 263
Sanitation on the Riviera, 3
Scarlatina (P.), 224
Scarlet Fever (C.), 95
Sciatica, Counter-irritants in (C.), 152
Scraps and Gleanings, 3?, 53, 71, 88, 109, 126, 144, 231, 284, 319, 351, 385, 404.
Scrotum, &c. (P.), 380
Seborrhœa and Baldness (P.), 189
Septic Infection in Operating Theatres, 91
Septic Treatment of Yellow Fever (C.), 80
Sinus Thrombosis (P.), 278
Skiagraphy (P.), 276
Skin Affections, The Diagnosis of Syphilitic (C.), 308
Skin Transplantation (C.), 378
Skull, Closure and Opening of the (P.), 258
Small-pox (P.), 242
Sociology. *See Modern Sociology*
Spinal Caries, Laminectomy in (P.), 188
Spine, Injuries of the (P.), 136
Spleen and Pancreas, Surgery of the (P.), 451
Spleen, Wandering (P.), 451
Splenectomy (P.), 451
"Star System" in Medical Societies, The, 425
Steaming instead of Cremation. 355
Stenosis, Cicatrical, of the Cardiac Orifice of the Stomach (P.), 276
Sterilisation by "Frying," 274
Stomach, The Complete Removal of the (C.), 256
Stomach, Dilatation of the (P.), 98, 450
Stomach, The Motor Activity of the (P.), 154
Stomach, Tumours of the (P.), 450
Stomach, Ulcer of the (P.), 451
Strabismus, Treatment of Ordinary Forms of (P.), 294
Strikes, The Cost of, 147
Student of Medicine, The, 16, 34, 54, 72, 88, 110, 126, 162, 178, 194, 214, 232, 265, 284, 320, 352, 370, 386, 422, 440, 458
Sub-conjunctival Injections (P.), 294
Subphrenic Abscess (C.), 430
Suicide, The Increase of, 113
Sunday Funds and Nursing Associations *See* p. 53
Sunstroke (P.), 343, (C.) 448
Suppuration, A Test for (P.), 2?6
Surgery, Abdominal (P.), 11
Surgery of the Brain (P.), 312
Surgery, Cerebral (P.), 257, 277, 295
Surgery, General (P.), 205, 225, 243, 256
Surgery, Genito-Urinary (P.), 45, 64, 380, 397
Surgery of the Intestines (P.), 415, 431
Surgery of the Liver and Gall-Bladder (P.), 65, 331, 345
Surgery of the Œsophagus and Stomach (P.), 276
Surgery of the Peritoneum (P.), 259, 275
Surgery, Rectal (C.), 327
Surgery of the Spine (P.), 188
Surgery of the Spleen and Pancreas (P.), 451
Surgery of the Stomach, 276
Swansea Hospital, The (W.), 71
Symblephoron, Obviating Recurrence after Operation for (P.), 294
Syphilis (P.), 398
Syphilis, Intra-muscular Injections of Mercury in the Treatment of (C.), 377
Syphilitic Skin Affection, The Diagnosis of (C.), 308, 326
Tetanus (P.), 312
Theatres, Churches, and Consumption, 251
Therapeutic Notes (C.), 360, 378, 396
Thyroid Extract for Backward Children (C.), 413
Tooth Brush, The, 113
Town v. Country, 165
Training the Sight, 388
Tuberculosis in the Insane, 269
Tuberculosis of the Kidney (P.), 45
Tuberculous Peritonitis (C.), 151, 184
Tumour, Cystic, of the Bladder (C), 117
Tumours of the Brain (P), 293
Tumours, Solid Mesenteric (P.), 416
Typhoid, Complications of (P.), 223
Typhoid, Endemic, 180
Typhoid, Secondary, 56
Typhoid Fever (P.) ,203
Typhoid Fever and London Water, 36
Typhoid Fever at Maidstone (C.), 7.
Typhoid Fever and its Prevention, 286
Typhoid Fever, Relapse in (C), 395
Typhoid Fever, Sequelæ of (C), 272
Typhoid Fever, The Outbreak of, at University College (C.), 45
Typhoid Fever, Vaccination Against, 74
Typhoid Ulcer, Perforating (P.), 433
Tyranny in Prison, 269
Ulcer of the Duodenum (P.), 187
Ulcer, Perforating Typhoid (P.), 439
Unqualified Assistant, The: The Other Side, 179
Unqualified "Medical Assistance, 163
Ureters, Catheterisation of (P.), 641
Ureters, Kidneys and (P.), 364
Urethra (P.), 397
Uric Acid (C.), 448
Urinary Apparatus, Suppuration of the (P.), 46
Urinary Calculi (P.), 364
Urine, Incontinence of (P.), 380
Urine, Retention and Extravasation of, in Children (C.), 431
Urology (P.), 328
Uterine Myomata, The Dangers of (C.), 377
Vaccination Bill, The, 442
Vaccination in Germany (C.), 292
Vaccine Lymph, Glycerinated, 215
Vaginal Douching after Parturition (C.), 60
Vaginal Incision, Early, in Septic Pelvic Disease (C.), 81
Varicose Veins and their Complications (C.), 25, 41, 94
Vertebræ, Traumatic Disease of the (P.), 137
Vesical Calculus (P.), 65, 380
Vesical Tumours (P.), 380
Vesico-Vaginal Fistula (P.), 156, 380
Vestries and Public Health, 197
Visceral Lesions (P.), 275
Vivisection Question, Our Position on the, 336
Vivisection Question, The *Star* on the (C.), 360
Voluntary Hospitals, *see* Growth and Scope
Vomiting while under Anæsthetics (C., 25, 41, 94
Water Supplies, The Local Government Board on Country (C.), 275
Water Supplies, The Pollution of, 146
Weak-sighted Children, The Initial Defect in, 40
Well Done *Pick-Me-Up*, 425
West African Fevers, 372
Westminster University, The Proposed, 165
What Do General Practitioners Want? 1
Whitlows (C.), 44
Whooping Cough, 443
Whooping Cough, Cases in Children's Hospitals, 387
Widal's Test for Typhoid Fever (P.), 204
Winter Quarters, 112
Woman's Reason, A (C), 171
Women's Work and Men's Wages, 181
Work for Cripples, 443
Workhouse Ambulance, The, 323
Workhouse Infirmaries, 233
Working Men and Hospitals (W.), 333
Workmen's Compensation Act, 1897, The (C.), 291
Wounded in Battle, The, 147
Wryneck, Spasmodic (C.), 118
Yarrow Convalescent Home, The, 381
Yellow Fever (P.), 256
Yellow Fever, Serum Treatment of (C.), 80
Zymotic Death Rate in 1897 (C.), 394

### THE SPECIAL SUPPLEMENT FOR NURSES.

Accidental Death, An, 127
African Health Resort, An, 120
All Saints Home for Trained Nurses, 36
American Letter, Our, 23, 71, 113, 156, 228
Antiseptics for Nurses, 163, 183, 199, 210, 217
Appointments, 9, 13, 21, 39, 50, 58, 71, 80, 85, 92, 106, 114, 121, 128, 141, 148, 155, 164, 174, 187, 194, 200, 214, 222, 233
Appointments, Minor, 8, 15, 29, 39, 50, 58, 63, 69, 80, 85, 98, 100, 116, 128, 141, 148, 155, 166, 174, 186, 194, 200, 214, 220, 225
Art of Fault-Finding, The 193
Beri-Beri, The Nursing of, 7
Beverages for Invalids, 8
Book and its Story, A, 47, 122, 132, 139, 157, 178
Book World for Women and Nurses, The, 8, 28, 49, 86, 166, 186, 194, 205, 221
Burns, The Late Mr. Walter Hayes, 75
Camberwell Scandal, The, 87
Centenary Celebration at the Royal Infirmary, Sheffield, 71
Children's Nurse, The, 202
Children's Nurse Again, The, 233
Christmas Entertainments, 129
Christmas Fare for Invalids, 101
Christmas Gifts, Our, 121
Christmas in Hospital, 120
Christmas Keeping, 138
Christmas Presents and Cards, 106
Common Sense, 115
Contrast, A, 33
Dangers of Nursing Syphilitic Cases, and How to Avoid Them, The, 93
Death in Our Ranks, 55, 139, 212
Diary of a New Pro., 219
Diet for Consumptive Patients, 154, 212
Diet for Sick Room, 212
Diet for Typhoid Fever Patients, 15
District Nursing in Plaistow and East Ham, 165
District and Parish Nurses, 37
Employment of Trained Nurses in Workhouses, On the, 204
Everybody's Opinion, 9, 16, 29, 38, 48, 57, 65, 79, 87, 97, 106, 115, 123, 132, 140, 149, 158, 168, 179, 187, 195, 205, 221, 234
Fashion in Nurses, A, 168
Funeral Customs in America, 55
Greek Ladies as Red Cross Nurses, 27
Gwelo Hospital, The, 64
Hampshire Unions in Conference at Winchester, 95
Help the N. rses to Help the Sick, 105
How Nurses May Help the Hospitals, 29
Hypnotism Across the Herring Pond, 121
Improvements at the London Hospital in 1897, Some, 114
In a Christmas Library, 103, 113
In Memoriam, 85
Invalid Cookery, 94
Irish Distressed Ladies' Fund, 65
Kodak Exhibition, The, 85
Lectures to Surgical Nurses, 3, 19, 43, 61, 76, 91, 119, 137, 153, 173, 191, 209, 225
Leper Hospital, In a, 148
London Temperance Hospital, 194
Man with a Brougham, The, 7
Management of Tracheotomy Cases, On, 44, 54
Marriage, 186
Mary Kingsley (Miss) at the Royal British Nurses' Association, 213
Massage. What is Massage? 220, 233
Meath Home for Epileptics, The, 45
"Members' Rights Defence Committee, The," 79
New and the Old Regulations at Guy's Hospital, The, 199
New Year in a New York Hospital, 100
News from the Nursing World, 1, 11, 17, 31, 41, 51, 59, 67, 73, 81, 89, 99, 107, 117, 125, 135, 143, 151, 161, 171, 181, 189, 197, 207, 215, 223
Notes on the Plague, 46
Notes and Queries, 10, 16, 30, 40, 50, 58, 66, 72, 80, 88, 98, 106, 116, 124, 134, 142, 150, 160, 170, 180, 188, 196, 206, 214, 222, 234
Novelties for Nurses, 9, 26, 39, 58, 71, 88, 96, 115, 173, 234
Nurse and Her Savings: A Dialogue, The, 24
Nurse's Visit to Texas, A, 48
Nurse's Wedding, A, 94
Nurses and Club Life, 21
Nurses for Klondyke, 228
Nurses at Maidstone: The Mayor's Reception, 102
Nurses for the Plague, 38
Nursing Appliances, 38
Nursing of Beri-Beri, The, 7
Nursing in China, 101
Nursing Directories, 96
Nursing of the Insane, The, 63
Nursing in Italy, 130
Nursing and Other Notes from Maidstone, 56
Nursing in Paris Hospitals, 175, 184, 192, 201, 211, 227
Nursing the Plague, 156, 203
Nursing in South Africa, 147
Nursing Syphilitic Cases, and How to Avoid Them, The Dangers of, 93
Nursing Typhus in Inniskea, 6
Operation in Private Houses, On Preparation for, 5
Plaistow Maternity Charity, 223
Practical Aspects of a Nurse's Life, 13, 33, 53, 69, 83, 109, 127, 145
Presentations, 9, 50, 63, 78, 123, 131, 142, 194, 205, 231
Prince of Wales's Hospital Fund for London 215, 232
Private Nursing Staff on New Lines, A, 193
Probationers for Madras, 109
Post-Graduate Clinics for Nurses, 4, 20, 34, 62, 70, 84, 110, 128, 138, 146, 164
Queen's Message to Miss Lumsden, The, 123
Queen Victoria's Jubilee Institute for Nurses, 129
Reading to the Sick, For, 10, 30, 40, 66, 72, 88, 116, 124, 142, 150, 160, 170, 177, 188, 196, 206, 214
Relations of the Public to the Nurse, The, 167, 176
Religious Influences in Nursing, 185
Retrospect, A, 39
Royal British Nurses' Association, The, 25, 35, 65, 77, 83, 92, 111, 148
Royal British Nurses' Association, Social Amenities of the, 115
Royal National Pension Fund for Nurses, 229
St. Clement's Maternity Nurses' Home, 167
Scharlieb, Mrs., at the Grosvenor Crescent Club, 154
Sensitiveness, 49
"She Hath Done What She Could," 131
Sick Diet: Milk, 200, 226
Temporary Nursing Arrangements at University College Hospital, 55
Tokens of Respect and Postponements, 53
Too Clever by Half, 186
Trained Nurses in Workhouses, On the Employment of, 204
Training School for Nurses, The, 218
Typhoid Fever at Maidstone, 37, 45
Victoria Commemoration Club, The, 26, 49, 80, 179
Wants and Workers, 13, 63, 70, 80, 85, 98, 106, 121, 131, 145, 164, 187, 193, 202, 209
Where to Go, 9, 27, 38, 50, 57, 63, 71, 78, 85, 92, 105, 113, 127, 141, 149, 159, 165, 193, 205, 219
Women as Hospital Dispensers, 46
Workhouse Infirmary Nursing Association, 56, 95
Workhouse Nurse, The, 146.
Workhouse Probationer, The, 155

Printed by W. H. and L. Collingridge, City Press, 148 and 149, Aldersgate Street, London, E.C.

### EARLY EDITION.

**Notices of Births, Deaths, and Marriages** are inserted in this column at a charge of 2s. 6d. for an announcement not exceeding four lines.

### NOTICE TO CORRESPONDENTS.

All MS., Letters, Books for Review, and other matters intended for the Editor should be addressed The Editor, "The Hospital," The Hospital Building, 28 & 29, Southampton Street, Strand, W.C.
The Editor cannot undertake to return rejected MS., even when accompanied by stamped directed envelope.

**Notice to Advertisers and Subscribers.**

All Advertisements, Orders for Copies of The Hospital, and Business Communications should be addressed to **THE MANAGER, (Not to The Editor),** The Hospital, The Hospital Building, 28 & 29, Southampton Street, Strand, London, W.C.

The Cost of Subscription, post free, is as follows:—Per week, 2½d.; per quarter, 2s. 8d.; per half-year, 5s. 3d.; per annum, 10s. 6d. Foreign:—Per annum, 15s. 2d.; payable, in all cases, in advance.

**NOTICE.—Vol. XXII. of "The Hospital" (April, 1897, to September, 1897), in handsome cloth binding, is now on sale at the Office of this Journal, price 7s. 6d. Cases for binding the Numbers contained in this Volume, 1s. 6d. Index to Vol. XXII., 2½d., post free.**
**The Monthly Part for October is now ready, Price 6d., by post 8d.**

### Notes and News.

### BOOKS RECEIVED.

Baillière, Tindall, and Cox.

"Hand Atlas Series." Vol. III., "Obstetric Diagnosis and Treatment," by Oscar Schaeffer, M.D., with 145 illustrations; Vol. IV., "Gynecology," by Dr. Oscar Schaeffer, with 173 coloured plate illustrations and 54 woodcuts.

"Lawson Tait's Perineal Operations." By W. J. Stewart McKay, M.B., M.Ch., B.Sc.

Isbister and Co.

"The Prophet's Mantle." By Christabel R. Coleridge.

J. and A. Churchill.

"Psilosis or Sprue." By George Thin, M.D.

"Economics, Anæsthetics, and Antiseptics in the Practice of Midwifery." By Haydn Brown, L.R.C.P., L.R.C.S.Edin.

Longmans, Green, and Co.

"Practical Domestic Hygiene." By J. Lane Notter, M.A., M.D., and R. H. Firth, F.R.C.S.

### The Student of Medicine.

### APPOINTMENTS.

A. Bethell, M.R.C.S.Eng, L.S.A., appointed Medical Officer of Health to the Bridgnorth Rural District Council. T. H. Chittenden, M.D., M.R.C.P., appointed Clinical Assistant to the Chelsea Hospital for Women, Fulham Road. B. O. Coates. M.D., appointed Clinical Assistant to the Chelsea Hospital for Women, Fulham Road. T. W. A. Daman, B.A.Cantab., M.B. and C.M.Edin., appointed Assistant House Surgeon to the County Hospital, Lincoln. H. E. Fraser, M.D.Edin., appointed Medical Superintendent to the Dundee Royal Infirmary. F. W. Grant, M.D.Edin., B Sc., C.M., appointed Medical Officer of Health for Lossiemouth and Branderburgh. W. H. Gravely, M.R.C.S.Eng., L.R.C.P.Edin., appointed Medical Officer for the Cowfold District of the Horsham Union. A. E. Hart, M.B., appointed Medical Officer for the Drypool District of the Sculcoates Union.

